# Patient Participation at Health Care Conferences: Engaged Patients Increase Information Flow, Expand Propagation, and Deepen Engagement in the Conversation of Tweets Compared to Physicians or Researchers

**DOI:** 10.2196/jmir.8049

**Published:** 2017-08-17

**Authors:** Audun Utengen, Dara Rouholiman, Jamison G Gamble, Francisco Jose Grajales III, Nisha Pradhan, Alicia C Staley, Liza Bernstein, Sean D Young, Kevin A Clauson, Larry F Chu

**Affiliations:** ^1^ Symplur Los Angeles, CA United States; ^2^ Stanford Medicine X Stanford University School of Medicine Stanford, CA United States; ^3^ Center for Social Innovation and Impact Investing Sauder School of Business University of British Columbia Vancouver, BC Canada; ^4^ Clinical Genetics Service Memorial Sloan Kettering Cancer Center New York, NY United States; ^5^ University of California Institute for Prediction Technology Department of Family Medicine University of California, Los Angeles Los Angeles, CA United States; ^6^ College of Pharmacy Lipscomb University Nashville, TN United States

**Keywords:** social media, patients, physicians, patient participation, congresses as topic, social networking, network analysis

## Abstract

**Background:**

Health care conferences present a unique opportunity to network, spark innovation, and disseminate novel information to a large audience, but the dissemination of information typically stays within very specific networks. Social network analysis can be adopted to understand the flow of information between virtual social communities and the role of patients within the network.

**Objective:**

The purpose of this study is to examine the impact engaged patients bring to health care conference social media information flow and how they expand dissemination and distribution of tweets compared to other health care conference stakeholders such as physicians and researchers.

**Methods:**

From January 2014 through December 2016, 7,644,549 tweets were analyzed from 1672 health care conferences with at least 1000 tweets who had registered in Symplur’s Health Care Hashtag Project from 2014 to 2016. The tweet content was analyzed to create a list of the top 100 influencers by mention from each conference, who were then subsequently categorized by stakeholder group. Multivariate linear regression models were created using stepwise function building to identify factors explaining variability as predictor variables for the model in which conference tweets were taken as the dependent variable.

**Results:**

Inclusion of engaged patients in health care conference social media was low compared to that of physicians and has not significantly changed over the last 3 years. When engaged patient voices are included in health care conferences, they greatly increase information flow as measured by total tweet volume (beta=301.6) compared to physicians (beta=137.3, *P*<.001), expand propagation of information tweeted during a conference as measured by social media impressions created (beta=1,700,000) compared to physicians (beta=270,000, *P*<.001), and deepen engagement in the tweet conversation as measured by replies to their tweets (beta=24.4) compared to physicians (beta=5.5, *P*<.001). Social network analysis of hubs and authorities revealed that patients had statistically significant higher hub scores (mean 8.26×10-4, SD 2.96×10-4) compared to other stakeholder groups’ Twitter accounts (mean 7.19×10-4, SD 3.81×10-4; t273.84=4.302, *P*<.001).

**Conclusions:**

Although engaged patients are powerful accelerators of information flow, expanders of tweet propagation, and greatly deepen engagement in conversation of tweets on social media of health care conferences compared to physicians, they represent only 1.4% of the stakeholder mix of the top 100 influencers in the conversation. Health care conferences that fail to engage patients in their proceedings may risk limiting their engagement with the public, disseminating scientific information to a narrow community and slowing flow of information across social media channels.

## Introduction

Traditionally, health care conferences are attended by experts, industry specialists, or others involved in fields specific to the conference in question. Health care conferences present a unique opportunity to network, spark innovation, and disseminate novel information to a large audience. Twitter is a microblogging and social media site with 313,000,000 monthly users, 82% of which are primarily mobile users. Twitter is gaining in popularity at health care conferences by allowing attendees to interact with one another and with their greater social networks, facilitating the sharing of information and ideas [1-13]. For example, the annual meeting of the American Society of Clinical Oncology saw an increase in tweets from 10,475 in 2012 to 44,034 in 2014 which resulted in 53,001,708 impressions in 2012 (ie, number of times the tweet was seen determined by the total number of followers who could view the tweet) and 154,362,922 impressions in 2014 [11].

We previously reported on the importance of including patients in medical conferences and identified four pillars of patient involvement in academic medical conferences [14]. These four pillars include accommodation (considering the physical needs of patients), codesign (patients codesign conference along with program creator), engagement (including patients in the audience and as presenters), and education and mentorship (guide patients toward conference stakeholder collaboration). By involving patients in health care conferences, a new voice is added to the discussion. Arguably, the ultimate purpose of health care conferences and health care is to improve the lives of patients and their families. By actively including patients in the conversation, patients are able to share their thoughts and express the issues that matter most to them [15]. Inclusion and engagement of patients can help drive information dissemination in health care conferences and widen research agendas to include new patient-centered domains.

Numerous studies have attempted to explain how patients and providers utilize and communicate via social media. In a systematic review of the literature, Smailhodzic et al [16] identified studies that examined patient and provider use of social media and identified six uses of social media for health-related purposes: emotional support, esteem support, information support, network support, emotional expression, and social comparison. Furthermore, the authors identified the primary effects of social media use by patients for health-related reasons, including positive effects (eg, empowerment, enhanced subjective well-being, enhanced psychological well-being, improved self-management and control) and negative effects (eg, diminished subjective well-being, loss of privacy, being targeted for promotion, and addiction to social media). The effects of patient use of social media on provider-patient relationships were also examined and included improved communication, harmonious relationships, and inferior interactions. In general, social media makes it easier to partner with patients and is their preferred method of communication.

Despite these studies, little is known about how stakeholders communicate via social media at health care conferences. To track information dissemination and diffusion during health care conferences, it is important to start by analyzing social networks. Social network analysis can be adapted to understand the flow of information between virtual social communities and to examine how individual user roles affect conversation dynamics [17]. Social networks are comprised of nodes and edges, with nodes representing individual users (represented as a circle) and edges representing connections between individual users (represented by a line). The degree of a node is the total number of edges connected to an individual node [18]. Furthermore, analysis of hubs and authorities within social networks may reveal additional information pertaining to that network. Authorities are defined as reputable sources of information that point to many hubs within a social network. Hubs are not authorities on their own, but point to multiple authorities within a social network. Hence, a good hub points to many good authorities, whereas a good authority is selected by a variety of good hubs. Connection topology of social networks is neither completely random nor completely systematic; it has characteristics of what Watts and Strogatz [19] first called “small-world networks,” in which the degree distribution of nodes approximates a power law distribution with pockets of cohesive communities throughout the network [19,20]. Health care conferences often have high community cohesion with quick access to information; however, information often does not disseminate to a broader audience. For a health care conference to disseminate information outside its community to a broader audience, “influential hub” nodes are essential. Influential hub nodes are social network users who, due to their position in the network, have shorter edges that connect them to other nodes/users in different communities [21]. Small network analysis can be utilized to demonstrate that engaged patients act as influential hub nodes during health care conferences and play an essential role in information dissemination. Engaged patients are broadly defined as any patient who actively participates in their health care through shared decision making, continued mindfulness of personal health needs within the context of their life, proactive seeking of information pertaining to their health, the setting of personal health goals, and the seeking of resources to achieve set goals [22].

In this study, we conducted a retrospective analysis of more than 7.5 million tweets from 1672 health care conferences that occurred from 2014 to 2016, which were registered in the largest online directory of health care conferences using social media [23]. We assessed three primary measures of social media performance concerning information dissemination during live health care conference coverage which included information flow, information propagation, and engagement in conversation in six stakeholder cohorts (patients, physicians and researchers, nonphysician health care professionals [HCPs], journalists, other health care individuals, and pharmaceutical organizations) and assessed performance of these cohorts against one another by these measures (Table 1). Definitions of stakeholder groups are presented in Table 2.

**Table 1:** Twitter metrics description.

Metric	Description	Purpose
Information flow	Total number of tweets as a performance indicator	Frequency of information disseminated during a health care conference
Engagement in conversation	Number of replies as a quality indicator	Measure of engagement and active conversation
Information propagation	Total number of potential impressions as dissemination network size	Prediction of network size; how many people/groups received your message?

**Table 2:** Definitions of stakeholder groups.^a^

Stakeholder	Definition
Patient	A person whose primary use of Twitter is to express their point of view as a patient with a specific disease or condition
Physicians and researchers	Those believed to be licensed MDs, DOs, or PhDs who bill directly for services, including residents and persons who work in the field of health-related research and/or academia
Health care professionals (HCPs)	Those believed to be health care professionals (eg, nurses, dietitians, respiratory therapists, nurse practitioners, pharmacists)
Journalists	Person whose profession is journalism or other news-related media
Other health care individual	Person working in the health care industry in a nonclinical role
Pharmaceutical organization	All organizations in the pharmaceutical industry

^a^ As defined by Symplur.

## Methods

### Categorization of Conferences and Stakeholders

Data was collected with the Symplur Signals research platform (Los Angeles, CA, USA) with direct access to the Twitter application program interface (San Francisco, CA, USA) [24]. We analyzed 7,644,549 tweets from 1672 health care conferences registered in Symplur’s Health Care Hashtag Project (the world’s largest collection of publicly available health care hashtags) from 2014 to 2016 (a total of 5692 conference hashtags), with at least 1000 tweets [23,25]. Metrics used in this study are defined in Table 1. The social network was analyzed to create a list of the top 100 influencers by mention from each conference. Influencers were subsequently identified and categorized by stakeholder group: patients, physicians and researchers, HCPs, journalists, other health care individual or pharmaceutical organizations based on Twitter accounts that publicly self-categorized their biographical description as certain stakeholders (eg, “radiologist,” “professor,” “nurse”; Table 2).

Multivariate linear regression models were created using stepwise function building to identify factors explaining variability as predictor variables for the model in which conference tweet were taken as the dependent variable. The categorization process involved a multinomial logistic regression multiclass classification model with a manual verification step, by which 156,149 Twitter accounts were categorized.

### Statistical Analysis

#### Comparative Analysis

Statistical analysis was conducted using the open source programing language R, version 3.3.3 (Vienna, Austria, 2017) [26]. A Welch two-sample *t* test was used to compare the relative performance between those conferences with at least one patient among its top 100 influencers by mentions and those conferences without any patients among its influencers, with the number of total tweets from the respective conferences as the information flow indicator.

#### Predictive Analysis

Multiple regression analysis was constructed to test if the performance metrics significantly (*P*<.05) depended on number of patients among the top influencers of a conference.

#### Number of Tweets, Replies, and Impressions

Multiple regression analysis was used to test if the health care stakeholders’ composition significantly predicted the conferences information flow (ie, number of tweets), engagement in conversations on Twitter (ie, number of replies), and information propagation from conferences on Twitter (ie, impressions). Replies are indicators of engagement in conversation tweets, which represent a quality, back-and-forth conversation and not simply a broadcast tweet or a random retweet. Information propagation was calculated based on total potential impressions by multiplying the number of tweets from each Twitter account with their number of followers then taking the sum of that number for all accounts tweeting during the conference.

#### Social Network Analysis (Hubs and Authorities)

Stanford Medicine X is an annual health care conference on emerging technology and medicine, focusing on patient-centered innovation and embraces the philosophy of Everyone Included, which places value on the voices of all health care stakeholders [27]. The influential hubs and authorities of the entire social conversation from the 2016 Stanford Medicine X conference was investigated by using the weighted hyperlink-induced topic search (HITS) algorithm. The values of the weighted HITS hub and authority for each Twitter participant as a node in this social network were calculated [28]. HITS was originally introduced by Kleinberg [21] to rate the importance of a node in a complex directed network using authority and hub values, where hub vectors y=(y_1_,...,y_n_)^t^ and authority vectors x=(x_1_,...,x_n_)^t^ are defined as:

x(t+1)=c(t)A
^t^y(t)

y(t+1)=d(t)Ax(t+1)

To shed more light on why patients bring better social performance to conferences, patient nodes were compared to nonpatient nodes using a Welch two-sample *t* test for both the hub scores and authority scores.

## Results

### Health Care Hashtag Project Conferences

Table 3 displays the total number of conferences in 2014, 2015, and 2016 registered with the Health Care Hashtag Project. During the 3 years included in the study (January 1, 2014 to December 31, 2016) a total of 5692 conferences were identified that utilized the Health Care Hashtag Project. Conferences with at least 1000 tweets were elected for analysis, which yielded 7,644,549 tweets from 1672 conferences, of which 749 had at least one patient in the top 100 influencers by mentions.

### Conference Stakeholders

**Figure 1:**
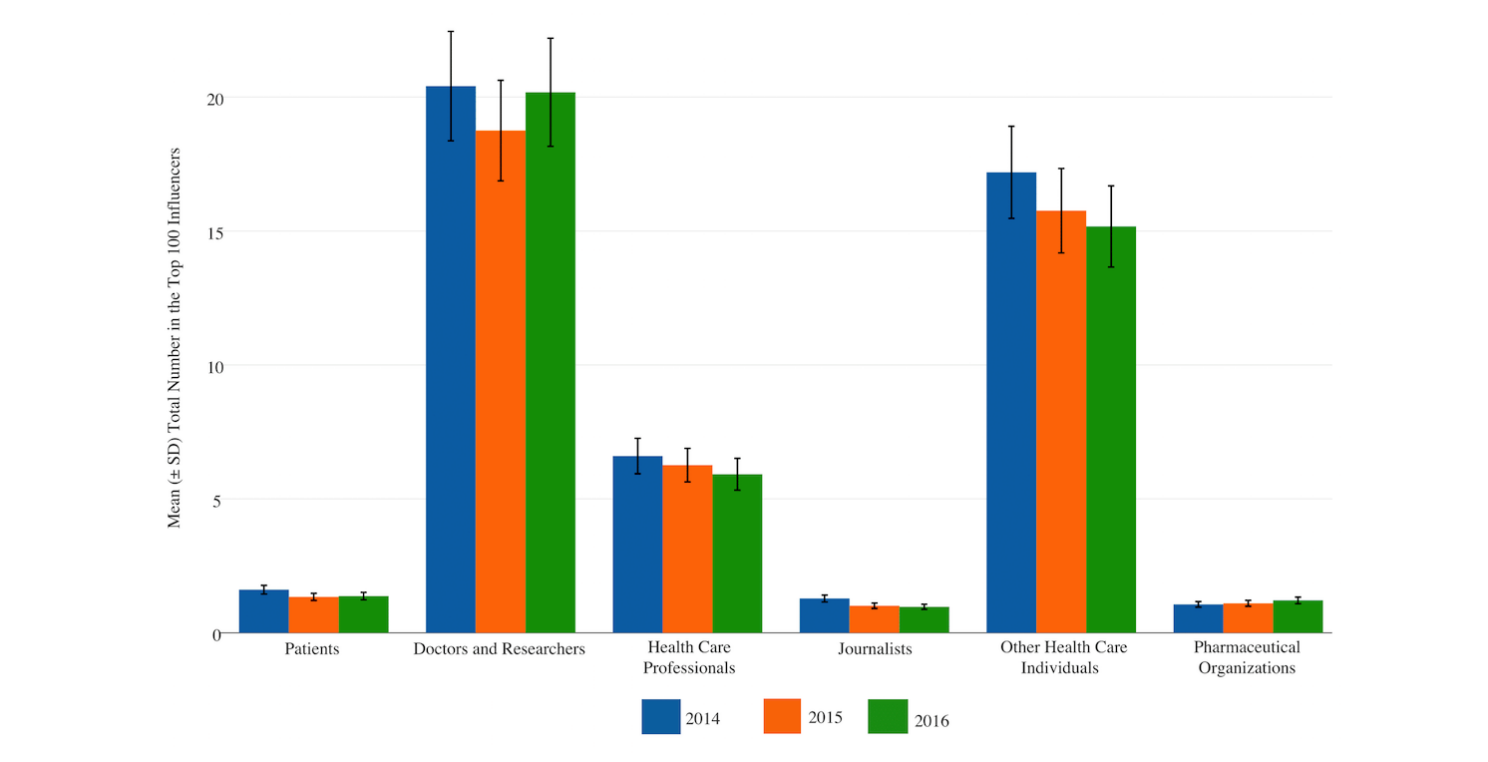
Stakeholder groups among top 100 influencers at health care conferences.

A total of 156,149 Twitter accounts were categorized into 16 stakeholder groups, of which 75,720 belonged to either a patient (n=2355), physician or researcher (n=32,930), HCP (n=10,344), journalist (n=1756), other health care individual (n=26,428), or pharmaceutical organization (n=1907) within the top 100 influencers by mention. Although 16 stakeholder groups were categorized, only six were isolated for analysis. Figure 1 and Table 4 show descriptive statistics of the top 100 influencers by mention within the six primary stakeholder groups analyzed. From 2014 to 2016 each category saw a decrease in the mean number of top 100 influencers by category with the exception of pharmaceutical organizations, which saw a mean increase of 0.15 (Table 4). From 2015 to 2016, the categories of patients, physicians and researchers, and pharmaceutical organizations saw a mean increase of 0.03, 1.43, and 0.11, respectively.

**Table 3:** Conferences registered with the Health Care Hashtag Project.

Conference metric	Year, n		
	2014	2015	2016
Total conferences	1428	1982	2282
Conferences with >1000 tweets	347	620	705
Total tweets from analyzed conferences	1,543,862	2,710,012	3,390,675

**Table 4:** Descriptive statistics for stakeholder groups among top 100 influencers at health care conferences.

Stakeholder and year		Mean (SD)	Median (range)
**Patients**			
	2014	1.61 (3.23)	1 (0-39)
	2015	1.34 (2.89)	0 (0-28)
	2016	1.37 (3.70)	0 (0-51)
**Physicians and researchers**			
	2014	20.41 (17.21)	16 (0-69)
	2015	18.75 (16.18)	13 (0-72)
	2016	20.18 (16.43)	15 (0-72)
**Health care professionals**			
	2014	6.60 (11.24)	2 (0-68)
	2015	6.26 (9.98)	2 (0-65)
	2016	5.92 (9.57)	2 (0-61)
**Journalists**			
	2014	1.28 (1.64)	1 (0-39)
	2015	1.01 (2.17)	0 (0-28)
	2016	0.97 (1.66)	0 (0-51)
**Other health care individuals**			
	2014	17.19 (11.34)	15 (0-68)
	2015	15.76 (10.05)	13 (0-51)
	2016	15.17 (9.57)	13 (0-51)
**Pharmaceutical organizations**			
	2014	1.06 (2.49)	0 (0-16)
	2015	1.10 (2.54)	0 (0-16)
	2016	1.21 (2.75)	0 (0-20)

### Comparison of Performance Between Conferences

**Figure 2:**
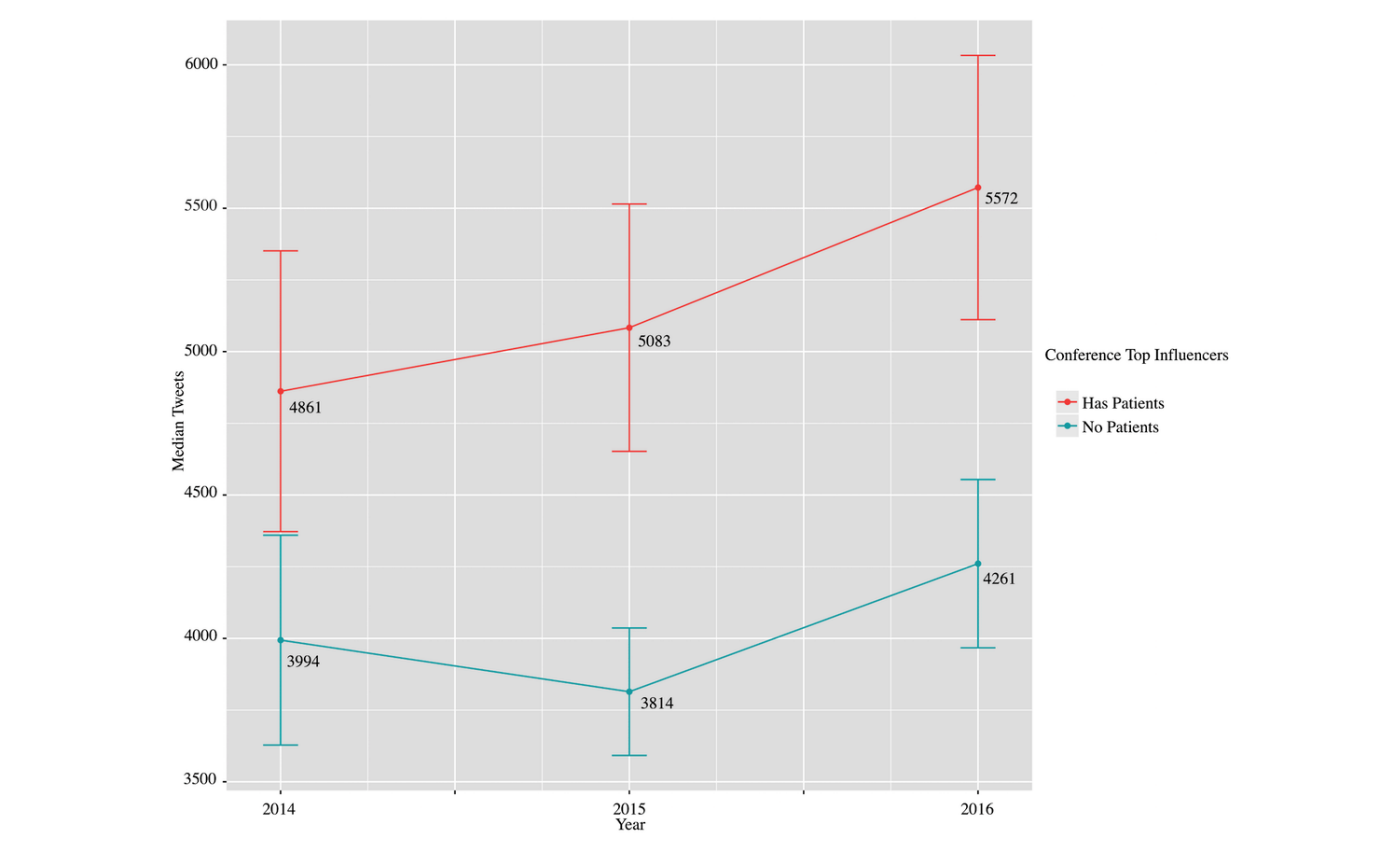
Comparison of health care conferences with patients in the top 100 influencers by mention and those without.

A combined analysis of the 2014, 2015, and 2016 conferences revealed that conferences with patients were found to have statistically significant higher number of tweets (mean 5222, SD 7320) compared to conferences that had no patients (mean 4044, SD 5108: *t*_1292.3_=3.7271, *P*<.001). The years 2015 and 2016 had statistically significant differences in the means between conferences with and without patients, although no statistically significant difference was found for 2014 (Figure 2). In 2014, conferences with patients had a mean total number of tweets of 4861 (SD 6604) compared to mean 3994 (SD 4700) for conferences with no patients (*t*_1.42_=327.05, *P*=.15). In 2015, conferences with patients had a mean total number of tweets of 5083 (SD 7122) compared to mean 3814 (SD 4144) for conferences with no patients (*t*_2.62_=411.34, *P*=.009). In 2016, conferences with patients had a mean total number of tweets of 5572 (SD 7913) compared to mean 4261 (SD 5941) for conferences with no patients in the top 100 influencers (*t*_2.40_=519.41, *P=*.01).

### Health Care Stakeholder Composition (Information Flow)

Multiple regression analysis was used to test if health care stakeholders’ composition significantly predicted the conferences information flow, a performance metric based on the number of tweets. The regression for 2014 to 2016 indicated that the six predictors explained 21% of the variance (*R*^2^=.21, *F*_6,1665_=73.35, *P*<.001). The number of patients among the top influencers significantly predicted better performance for the conferences (beta=309, *P*<.001), as did the number of physicians and researchers (beta=138, *P*<.001), HCPs (beta=118, *P*<.001), journalists (beta=440, *P*<.001), other health care individuals (beta=130, *P*<.001), and Twitter accounts representing pharmaceutical organizations (beta=693, *P*<.001). For every increase of one patient among the top 100 influencers by mention, the conference’s predicted number of tweets increased by 309.

### Health Care Stakeholder Composition (Engagement in Conversation)

The results of the regression analysis for the combined years of 2014 to 2016 indicated the six predictors explained 24% of the variance (*R*^2^=.24, *F*_6,1665_=85.51, *P*<.001). The number of patients among the top influencers significantly predicted better engagement in conversations for the conferences (beta=25, *P*<.001), as did the number of physicians/researchers (beta=6, *P*<.001), HCPs (beta=5, *P*<.001), journalists (beta=12, *P*<.001), other health care individuals (beta=6, *P*<.001), and Twitter accounts representing pharmaceutical organizations (beta=8, *P*<.001).

### Health Care Stakeholder Composition (Information Propagation)

The results of the regression analysis for the combined years of 2014 to 2016 indicated the six predictors explained 18% of the variance (*R*^2^=.18, *F*_6,1665_=59.49, *P*<.001). The number of patients among the top influencers significantly predicted larger audience and wider potential spread of information for the conferences (beta=1,781,222, *P*<.001), as did the number of physicians/researchers (beta=261,253, *P*<.001), journalists (beta=2,669,759, *P*<.001), other health care individuals (beta=261,162, *P*<.001), and Twitter accounts representing pharmaceutical organizations (beta=2,819,703, *P*<.001).

### Social Network Analysis (Hubs and Authorities)

**Figure 3:**
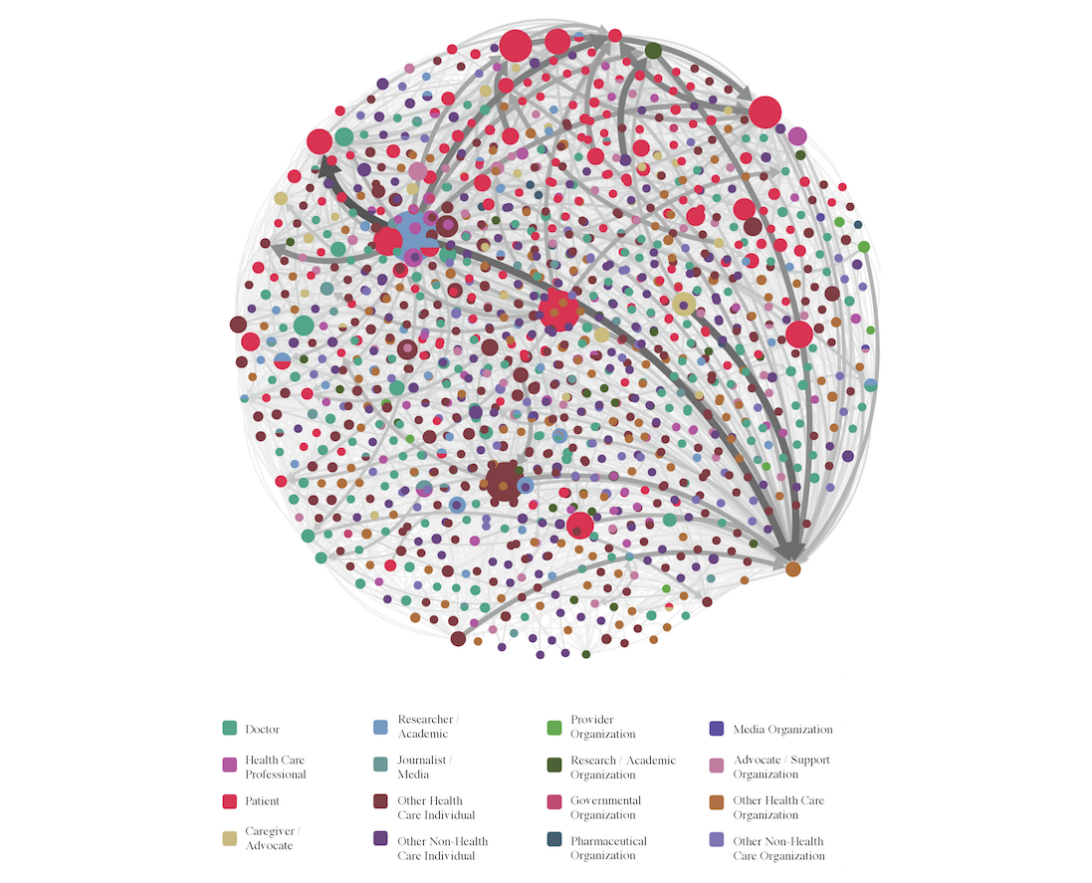
Social network analysis of the 2016 Stanford Medicine X conference based on hubs and authority score.

Social network analysis of hubs and authorities revealed that patients were found having statistically significant higher hub scores (mean 8.26×10^-4^, SD 2.96×10^-4^) compared to Twitter accounts not owned by patients (mean 7.19×10^-4^, SD 3.81×10^-4^; *t*_273.84_=4.302, *P*<.001). There were no statistically significant differences in the authority scores between patient and nonpatient Twitter accounts (Figure 3).

## Discussion

During health care conferences, engaged patients use their position in social networks as an influential hub node to disseminate information to a broader community beyond the conference network. Engaged patients with firsthand experience of a chronic condition contribute their expertise by asking essential questions that lead engaging conversations as measured by increased patient activation levels [29]. The role of the engaged patient goes beyond information diffusion; they add value to the conversations by either questioning the reported information or supporting it based on their personal experiences and expertise. In this study, we demonstrated that engaged patients are more effective than physicians or researchers in three primary measures of social media performance concerning information dissemination during live health care conference coverage. Between the years of 2014 and 2016, 5692 health care conferences with specific Twitter feeds whose hashtags were registered with Symplur’s Hashtag project were identified. Each consecutive year saw an increase in registered hashtags. Through analysis of health care conference hashtags, we identified individual conference stakeholders to determine the extent to which patients facilitate the propagation of information flow out of health care conferences via Twitter.

True patient engagement is based on more than just the raw number of tweets that patients contribute but rather on thoughtful tweets, quality replies, meaningful conversations, and exchange of knowledge facilitated by engaged patients and other health care conference stakeholders. Although the total number of tweets at health care conferences increased from 2015 to 2016, our results indicate that conferences that include patients have a significantly higher number of tweets than conferences that do not include patients (beta=309, *P*<.001). Inclusion of engaged patients in health care conferences increases the estimated number of impressions by beta=1,781,222 (*P*<.001); however, what is valuable about inclusion of engaged patients is their effect on increase of quality of conversation. Engaged patients increase the number of quality tweets by beta=25 (*P*<.001), double the impact of any other stakeholder group. Although patients are significant expanders of health care tweet propagation and accelerate information flow out of health care conferences, they make up 1.4% of the stakeholder group in the top 100 influencers of the conversation.

Social network analysis of hubs and authorities provided evidence that patients are functioning as hubs within Twitter at health care conferences to a larger degree than are nonpatients. In the context of this social conversation, authority values are large for nodes with significant incoming mentions and conversations from large hub nodes, and hub values are large for nodes with significant outgoing mentions and conversations to high-authority nodes. Patients actively engage authorities acting as good hubs and may be seen as a social glue that in itself encourages and creates even more engagement from nonpatients as reflected in our finding for the performance metric number of tweets. By failing to include patients, health care conferences risk attenuated engagement with the public, disseminating scientific information to a narrow social network and reducing the speed of information flow across social media channels, which ultimately deters from the academic missions of health care conferences.

Our results suggest that when health care conferences include patients as conference stakeholders, patients influence the conference Twitter conversation by increasing total number of tweets and increasing spread of conference information across social networks, which will yield better social media performance outcomes for the conference. Strategies such as those outlined by the European Patients’ Forum, the patient advocate-originated Cinder Blocks movement, and the Everyone Included initiative facilitate an environment in which patients are trusted, respected, and are appreciated for the expertise they bring to the conversation, openness and experimentation are normal and expected, patients have personal ownership of issues in health, individual patient stories have global impact, and patient voices and choices are incorporated into stakeholder decisions and actions [30-32].

By utilizing patient inclusion frameworks such as those just described, conference developers can build trust and respect with patient populations, create a shared mindset for change, better identify issues that matter most to patients, produce more innovative and creative solutions to health problems, and create a shared, inclusive culture of health. Patient inclusion initiatives have been implemented by the Outcome Measures in Rheumatology (OMERACT) conferences since 2002, where now more than 10% of conference participants are patients [33]. Since including patients as conference stakeholders, OMERACT has identified novel outcome measures important to patients and has incorporated the perspective of patients into the development of novel outcome measures [33-35]. Engaging patients in health care conferences may occur via other methods of multimedia delivery, such as video live streams, bringing the conference directly to patients who may not be able to attend the conference in person but still have thoughts to share.

Patients not only expand information out of health care conferences but also feed knowledge back in by sharing personal experiences and voicing issues that matter to them. This concept is again illustrated through the OMERACT conferences, which after including patients as conference stakeholders, redeveloped their research agenda and developed novel clinical trials based on outcomes that patients identified as the most relevant to their health and quality of life [34]. By including patient voices, conferences such as OMERACT and Stanford Medicine X have increased physicians’ knowledge by showing them what it is like to live with a disease in which they are trained to treat [27]. By giving patients a voice at health care conferences, they act as both educators and participants, facilitating discussions and engaging providers by sharing stories and ideas, which acts to widen existing research agendas [36-38].

Some may believe that not all health care-related conferences are pertinent for patient inclusion, such as health care conferences hosted by professional medical societies that are intended to garner continuing medical education units (CMEs) or continuing education units for its members. We believe that patient inclusion in these types of conferences is pertinent, and does benefit both the specific professional medical society hosting the event, and the broader medical community at large. Furthermore, in July of 2016, the Accreditation Council for Continuing Medical Education announced new criteria for accreditation with commendation that incorporates the inclusion and engagement of patients in the planning and delivery of CMEs as planners and faculty in the accredited conference or program [39].

By incorporating patient inclusion frameworks in health care conferences and research, future studies should strive to recognize and include patients as valuable team members who bring novel expertise into the conversation. Patient inclusion frameworks, such as those previously discussed, may be used to facilitate multidisciplinary and interprofessional collaboration in health care and scientific research expanding possible outcome measures. Furthermore, by recognizing and including patients as stakeholders, health care conferences can effectively spread information through social media to new nodes reaching a broad and unique audience. The inclusion of engaged patients leads to higher tweet volume out of health care conferences and facilitates the feeding of knowledge back in via patient expertise, experience, and opinion. By including patient voices, the traditional method of scientific inquiry can be expanded, accelerated, and powered leading to novel research questions and unique patient-centric outcome measures. The simple act of including patients in health care conferences has the potential to revolutionize medical research by shifting focus toward patient-identified issues that may otherwise be overlooked by HCPs and researchers.

There are several limitations of this study. Stakeholder role was determined by self-reported information on Twitter user’s personal biographies, which may not be completely accurate. For example, Twitter profiles may underreport patient status whereas others may belong to multiple stakeholder groups, such as an account belonging to an individual who is both a HCP and a patient, giving them a unique perspective. Due to the large amount of data that was collected, it was not possible to analyze tweet content or tweets that were shared or liked by others in the network. Another limitation is that our social network analysis was only based on one health care conference, the 2016 Stanford Medicine X conference, which has upwards of 10% patients as stakeholders.

Future studies should perform network analyses comparing conferences that do and do not include patients among the top 100 stakeholders by mention. Furthermore, future studies should examine *k*-core decomposition, the largest subgraph in which vertices have a minimum of *k* interconnections, of health care conference social networks among conferences that do and do not engage patients and whether the number of patients included in a conference affect *k* scores [40-42].

## Abbreviations

CME: continuing medical education
HCP: health care professional
HITS: hyperlink-induced topic search
OMERACT: Outcome Measures in Rheumatology
